# Exploring the Molecular Interactions of Symmetrical and Unsymmetrical Selenoglycosides with Human Galectin-1 and Galectin-3

**DOI:** 10.3390/ijms23158273

**Published:** 2022-07-27

**Authors:** Luciano Pirone, Ferran Nieto-Fabregat, Sonia Di Gaetano, Domenica Capasso, Rita Russo, Serena Traboni, Antonio Molinaro, Alfonso Iadonisi, Michele Saviano, Roberta Marchetti, Alba Silipo, Emilia Pedone

**Affiliations:** 1 Institute of Biostructures and Bioimaging, Consiglio Nazionale Delle Ricerche (CNR), 80134 Naples, Italy; luciano.pirone@cnr.it (L.P.); digaetan@unina.it (S.D.G.); rita.russo@unicampania.it (R.R.); 2 Department of Chemical Sciences, University of Naples Federico II, Via Cinthia 4, 80126 Naples, Italy; ferran.nietofabregat@unina.it (F.N.-F.); serena.traboni@unina.it (S.T.); molinaro@unina.it (A.M.); iadonisi@unina.it (A.I.); roberta.marchetti@unina.it (R.M.); 3 Interuniversity Research Centre on Bioactive Peptides (CIRPEB), University of Naples Federico II, 80134 Naples, Italy; domenica.capasso@unina.it; 4 Center for Life Sciences and Technologies (CESTEV), University of Naples Federico II, 80145 Naples, Italy; 5 Institute of Crystallography, Consiglio Nazionale Delle Ricerche (CNR), 81100 Caserta, Italy; msaviano@unina.it

**Keywords:** galectin, selenoglycosidic inhibitors, NMR

## Abstract

Galectins (Gals) are small cytosolic proteins that bind β-galactoside residues via their evolutionarily conserved carbohydrate recognition domain. Their dysregulation has been shown to be associated with many diseases. Consequently, targeting galectins for clinical applications has become increasingly relevant to develop tailored inhibitors selectively for one galectin. Accordingly, binding studies providing the molecular details of the interaction between galectin and inhibitor may be useful for the rational design of potent and selective antagonists. Gal-1 and Gal-3 are among the best-studied galectins, mainly for their roles in cancer progression; therefore, the molecular details of their interaction with inhibitors are demanded. This work gains more value by focusing on the interaction between Gal-1 and Gal-3 with the selenylated analogue of the Gal inhibitor thiodigalactose, characterized by a selenoglycoside bond (SeDG), and with unsymmetrical diglycosyl selenides (unsym(Se). Gal-1 and Gal-3 were produced heterologously and biophysically characterized. Interaction studies were performed by ITC, NMR spectroscopy, and MD simulation, and thermodynamic values were discussed and integrated with spectroscopic and computational results. The 3D complexes involving SeDG when interacting with Gal-1 and Gal-3 were depicted. Overall, the collected results will help identify hot spots for the design of new, better performing, and more specific Gal inhibitors.

## 1. Introduction

Galectins (Gals), members of the lectin family, are small cytosolic proteins that bind β-galactoside residues through their evolutionarily conserved carbohydrate recognition domain (CRD) [1]. To date, 15 members of the galectin family have been identified in mammals and have been named following the sequential order of discovery [2]. Galectins are classified into three groups according to their CRD organization. Prototype galectins (including Gal-1, -2, -5, -7, -10, -11, -13, -14, and -15) are homodimers in solution formed via a noncovalent interaction through their sole CRD while tandem-repeat galectins (Gal-4, -6, -8, -9, and -12) possess two distinct CRDs at their N- and C-termini that are connected by a linker of variable length. The third group is represented only by Gal-3, which in its chimera structure displays a CRD at the C-terminus and a short non-lectin peptide motif (Gly–Pro–Tyr-rich domain) at the N-terminus.

Protein–carbohydrate interactions are crucial for the communication of biomolecules within and between cells. Since galectins are able to recognize glycoconjugates containing β-galactoside present on the cell surface, in extracellular matrices or in the lumen of intracellular vesicles, they are involved in a variety of biological processes, such as apoptosis, cell proliferation, or inflammation [2,3]. Consequently, their dysregulation has been associated with a wide range of diseases, such as cancer progression, autoimmune disorders, fibrosis, arthritis, obesity, cardiovascular diseases, allergies, and microbial infections [4,5]. As a result, targeting galectins for clinical applications has become an intense area of research and among them, Gal-1 and -3 are the best studied mainly for their roles in cancer progression. Since most of the activities of galectins are associated with their carbohydrate-binding characteristics, the inhibition of CRD by antagonists able to compete with the natural ligand seems to be a feasible option, not only to reveal their exact functions, many of which are still unexplored, but also to develop new molecules for therapeutic intervention. Different polysaccharide-based inhibitors have been developed and some of them are currently being evaluated in Phase I or Phase II clinical trials for various cancers [6,7]. In addition, the monovalent inhibitor TD139 (Galecto Biotech, Copenhagen, Denmark), showing an excellent affinity both to Gal-1 and Gal-3 (0.22 µM ± 0.05 for Gal-1 versus 0.068 µM ± 0.01 for Gal-3 from ITC determination) [4,5], has ended Phase Ib/IIa clinical trials for the treatment of idiopathic pulmonary fibrosis [8]. This molecule represents an optimized derivative of thiodigalactoside (TDG) with two identical substituents (4-fluorophenyl-triazole) at positions C3 and C3′ of the TDG. The replacement of the oxygen atom with a sulfur in TDG had already led to improvements in the enzymatic/hydrolytic stability, electing TDG as a starting scaffold for further derivatives. Successively, the introduction of selenium as the bridging atom in place of sulfur created a new version of diglycosylated analogues characterized by a selenoglycoside bond between two galactose units (SeDG) [9,10]. This modification proved to be a winning move not only because selenium has been incorporated into carbohydrates to assist in X-ray crystal structure determination using single/multiple wavelength anomalous diffraction techniques [11] and in NMR studies [12] but mainly due to its enhanced protease stability and its inherent antioxidant and peroxidant properties [10]. Furthermore, we have recently featured a new version of SeDG, introducing a lipophilic benzyl group at C-3 of both sugar residues [10] that was revealed to bind both Gal-3^CRD^ and Gal-9N^CRD^ and showed anti-proliferative and anti-migration effects on a melanoma cell line and anti-angiogenesis activities. Its relatively high affinity and its anti-migration and anti-angiogenesis properties pave the way for further development of such compounds as anti-tumor agents. 

The next challenging step will be to develop tailored inhibitors selectively targeting a specific galectin, and therefore able to distinguish and select within the galectin members. The differential activities of specific galectins in normal and pathological processes [1] further explain the urgent need to develop potent and selective inhibitors. The high similarity in CRD structures is clearly a main issue. Consequently, studies providing structural details of the interaction between galectins and ligands may be useful for the rational design of increasingly potent and selective inhibitors. In this regard, this work focuses on the interaction between Gal-1 and Gal-3 with SeDG and an unsymmetrical diglycosylated selenide bearing a galactose and an acetamido-glucose residue, unsym(Se) [9], assessed by ITC and analyzed by NMR and computational studies. If unsym(Se) is unable to properly accommodate in the binding site of both galectins, we can describe the recognition and binding process of SeDG with Gal-1 and Gal-3. We were able to depict the 3D complexes involving SeDG when it interacts with Gal-1 and Gal-3, describe the network of polar and hydrophobic interactions that stabilize the complexes, and highlight how the symmetric SeDG adopts two different bound conformations in the binding site of Gal-1 and Gal-3, thus indicating hot spots for the design of new, better performing, and more specific Gal inhibitors.

## 2. Results and Discussion

### 2.1. Biophysical Features of Gal-1 and Gal-3^CRD^

The expression and purification of Gal-3^CRD^ (amino acid residues 112–250) was carried out as described in Di Gaetano et al., 2022 [10]. The choice of the extension of the construct is dictated by the crystallization conditions reported in the literature. The gene encoding the full-length Gal-1 (residues 1–135) was cloned into the vector pETM11. *Escherichia coli* BL21(DE3)GOLD cells were transformed with recombinant construct and the expression of the recombinant protein was induced as described in the experimental section. The final yield was 20 mg/mL. 

**Figure 1:**
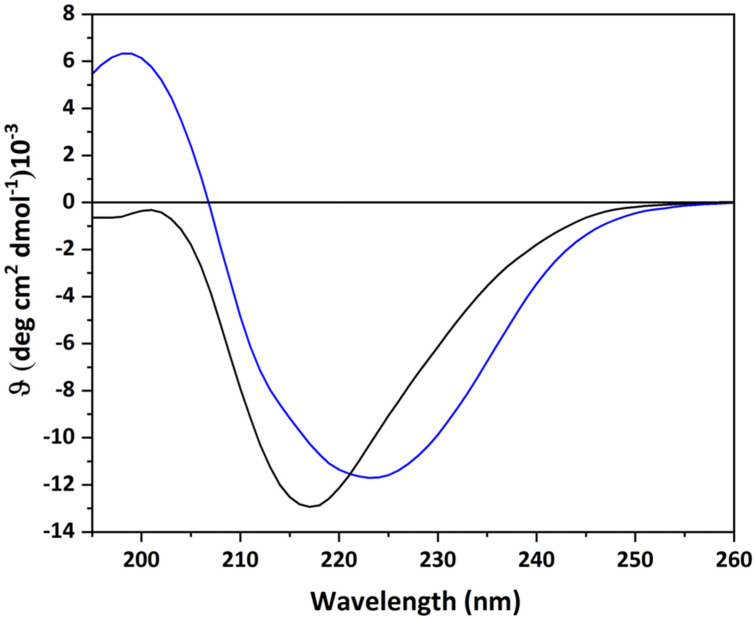
Far-UV CD spectra of Gal-1 (black line) and Gal-3^CRD^ (blue line).

To obtain insight into the structural integrity of the two recombinant proteins, the protein conformation was investigated by far-UV CD spectroscopy. For both proteins examined, the spectra revealed not only that the two proteins were correctly folded but also resembled a β-sheet-rich protein as expected for the secondary structures reported by X-ray crystallography (Figure 1) [8]. In fact, both spectra showed a single minimum around 220 nm, typical of the β-sheet structure.

However, comparing the spectrum of the proto-type human Gal-1 with the only member of the chimera type, Gal-3, significant differences were revealed, as already observed with the galectin domain of Gal-9 of the tandem-repeat-type group [13]. Such differences can result from the particular characteristics of the topological arrangement, such as the length of the filaments, intra/inter sheet twists, or β-turns.

The thermal stability of both recombinant proteins was also explored by far-UV CD spectroscopy. The Tm values derived from temperature ramp denaturation curves following the CD signal at 218 nm were about 65 °C for both proteins (Supplementary Materials Figure S1A,B), in agreement with the theoretical calculations from the crystallographic structures (for Gal-1 (PDB ID: 1GZW) and for Gal-3 (PDB ID: 4R9C)) and from experimental data [14,15]. As already verified, this fold is particularly stable as expected for the melting of a β-sheet structure [15].

**Figure 2:**
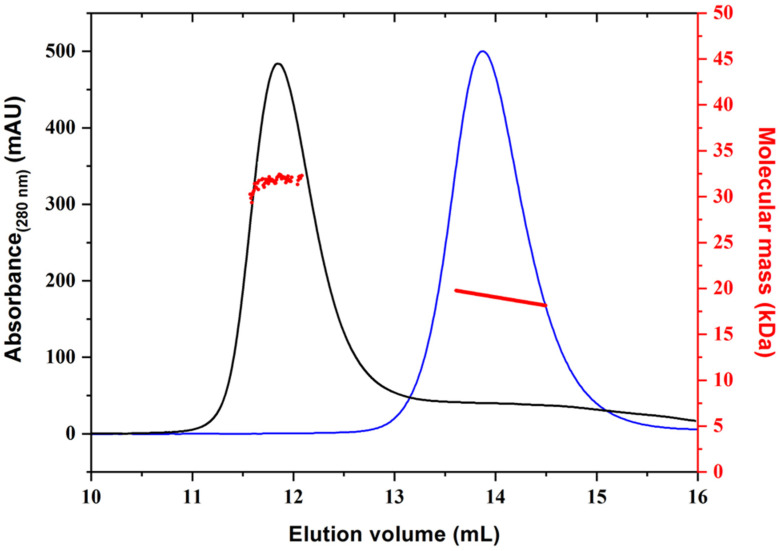
Light scattering measurements. The plots report the molecular mass and absorbance (280 nm) versus the elution volume for Gal-1 (black line) and Gal-3^CRD^ (blue line).

In addition, the oligomeric state of the proteins was assessed using light scattering measurements (Figure 2). This analysis demonstrated that while Gal-1 is dimeric in solution, Gal-3 is a monomer (Table 1), in perfect agreement with literature data [16].

### 2.2. Interaction Studies by ITC

**Figure 3:**
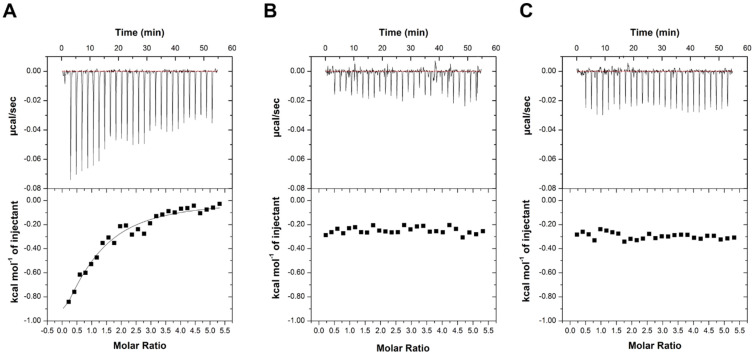
ITC binding studies on Gal-1. (**A**) Titration with SeDG and (**B**) with unsym(Se) in (**C**) the titration of buffer (negative control) is shown. The top panels of the graphs correspond to the injections while the bottom panels correspond to the integrations of the peaks according to the molar ratio of the tested molecules.

**Figure 4:**
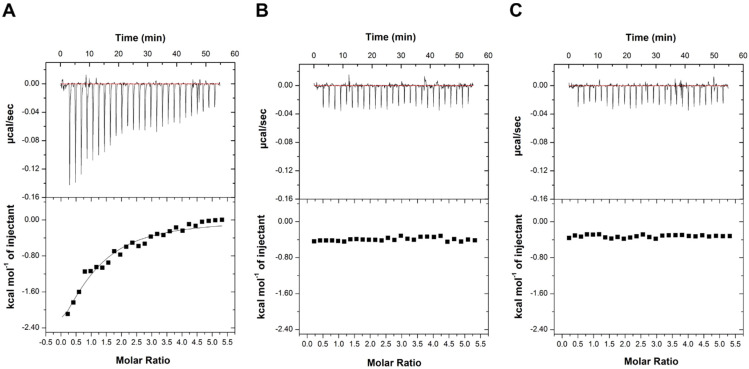
ITC binding studies on Gal-3^CRD^. (**A**) Titration with SeDG and (**B**) with unsym(Se) in (**C**) the titration of buffer (negative control) is shown. The top panels of the graphs correspond to the injections while the bottom panels correspond to the integrations of the peaks according to the molar ratio of the tested molecules.

Both proteins, once purified to homogeneity, were demonstrated to be fully active as they were able to bind lactose (Table 2, Supplementary Materials Figure S2 and ref. [10]). Both purified proteins were then probed for their binding affinity to SeDG and unsym(Se) by ITC. The K_D_ values obtained for Gal-3^CRD^ were 20.2 ± 2.2 µM, a value comparable with the K_D_ calculated for Gal-1 (23.7 ± 4.2 µM) (Figure 3 and Figure 4). The resulting values of K_D_, ΔG, ΔH, -TΔS, and n (stoichiometry) are summarized in Table 2. In all cases, the n-values indicate a 1:1 ratio of galectin/inhibitor. As observed for Gal-3^CRD^ and for Gal-1, the negative ΔH suggests an exothermic reaction between galectin and SeDG while the positive ΔS suggests entropically driven reactions involving hydrophobic interactions. It worth noting that the ΔH involved in the interaction between Gal-1 and SeDG is lower than that with Gal-3^CRD^, denoting that such a difference could be ascribed to the different number of contacts in the interaction interface region. On the contrary, both proteins showed no binding to the unsymmetrical molecule. 

### 2.3. NMR Binding Experiments/Analyses and Computational Studies

The interaction of the synthetized selenoglycosides SeDG and unsym(Se) with Gal-1 and Gal-3^CRD^ were described by means of ligand-based NMR techniques and computational approaches, with the aim of depicting the 3D complexes and their properties.

**Figure 5:**
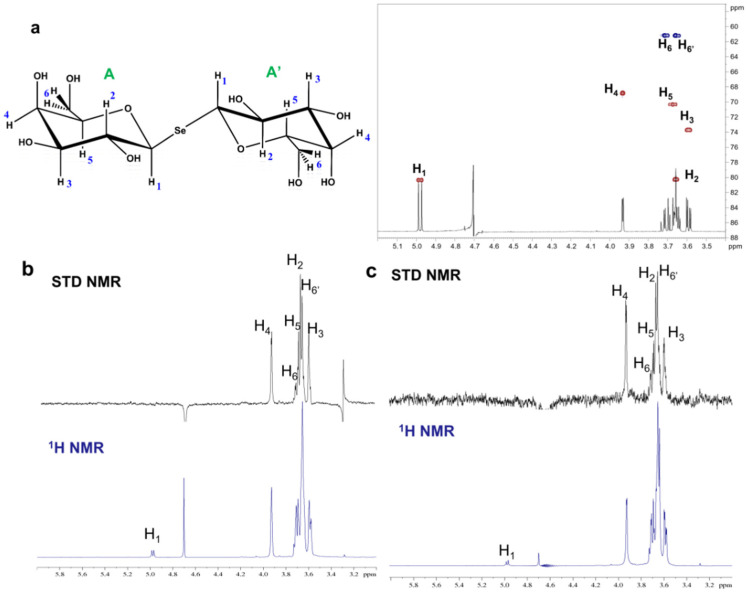
NMR analysis of SeDG with Gal-1 and Gal-3^CRD^. (**a**) The SeDG ligand chemical structure is colored according to the symbol-nomenclature for glycans (SNFG) and the HSQC NMR spectrum of the symmetric selenoglycoside with the resonances’ assignment. The galactose residues are named as A and A’. (**b**) ^1^H NMR reference spectrum (bottom) and 1D STD NMR spectrum (up) of the 1:40 mixture of Gal-3^CRD^: SeDG. (**c**) ^1^H NMR reference spectrum (bottom) and 1D STD NMR spectrum (up) of the 1:30 mixture of Gal-1: SeDG.

Saturation transfer difference (STD) NMR experiments were used to describe at the molecular level the recognition and binding processes of SeDG and unsym(Se) ligands with Gal-1 and Gal-3^CRD^. STD NMR analysis indicated that Gal-3^CRD^ and Gal-1 are both able to recognize SeDG, as suggested by the STD NMR enhancements observed for both mixtures Gal-3^CRD^:SeDG and Gal-1:SeDG (Figure 5). 

The spectra of the two systems appeared similar, with subtle but significant differences in the STD effects, including the highest STD signal (Supplementary Materials Tables S1 and S2, and Figure S3). Indeed, almost all protons of the Gal units of SeDG showed STD enhancements, with the sole anomeric proton H1 giving no STD signal, indicating that the anomeric positions of both Gal units were not involved in the interaction. Nevertheless, the signal overlapping between the protons H2, H5, and H6′ partially impaired a fine attribution of their contribution to the binding and, therefore, the evaluation of the strength of the interaction. Moreover, a further shortcoming was encountered in the attempt to design the epitope mapping, namely the symmetry of the ligand. For example, focusing on proton H3, due to the symmetry of the complex, there was no way to assess whether its interaction occurred via both Gal sugars, or if only H3 of one of the subunits was involved in the interaction. To overcome this issue, the STD NMR results were combined with computational approaches that allowed description of the epitope mapping and the proposal of a 3D complex for both systems, as described below. 

When working with the unsym(Se) ligand, no STD NMR effects were observed, meaning that there was no interaction between unsym(Se) and Gal-1 or Gal-3^CRD^, with the results being consistent with the above-mentioned ITC interaction results. Moreover, further computational studies were also in agreement, since in both cases (unsym(Se) interacting with Gal-1 and Gal-3^CRD^), the *N*-acetyl moiety at position 2 experienced steric hindrance with both galectins when accommodating the ligand in the binding pocket (Supplementary Materials Figure S4).

### 2.4. Computational Studies: 3D Complexes of SeDG with Gal-3^CRD^ and Gal-1

Computational studies were performed by combining docking calculations, carried out with Autodock 4.2 [17], and molecular dynamics (MD) simulation, carried out with AMBER18 [18]. Due to the peculiarity of the ligands, the parametrization of the selenium glycoside was necessary to run the MD simulations with AMBER, where the parameters for the Se atom were missing. Therefore, we chose to carry out a first docking calculation by modeling the binding pocket of Gal-3 (PDB ID: 4R9C) and Gal-1 (PDB ID: 1GZW) with a ligand in which the Se atom was substituted with an oxygen atom (identified from here on as Sym(O) ligand). The lower energy family (Supplementary Materials Figure S5) was the most populated for both complexes (with Gal-1 and Gal-3^CRD^) and the obtained representative docked poses were, therefore, used as the starting point for the MD simulations with the Sym(O) ligand. In both cases, the ligand was stable in the binding pocket for the entire simulation time.

**Figure 6:**
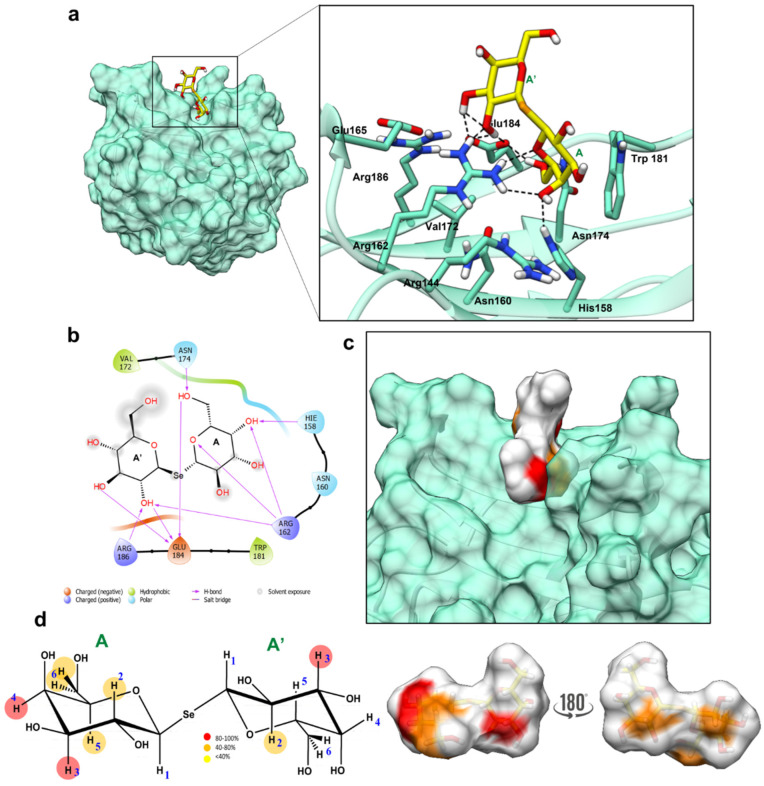
Interaction between Gal-3^CRD^ and SeDG. (**a**) The 3D model of the Gal-3^CRD^—SeDG. Close-up view of a representative frame from the most populated MD cluster with the main residues involved in the binding depicted as sticks. (**b**) Two-dimensional plot representing the interactions between the SeDG and Gal-3^CRD^ binding pocket residues. (**c**) Gal-3^CRD^/SeDG 3D complex as derived by the combination of the MD and STD-NMR results. The ligand surface is colored according to the STD enhancements. (**d**) Interacting epitope map of SeDG derived from STD-NMR data on the left and 3D representation of SeDG in the bioactive conformation with the molecular surface colored according to the STD enhancements.

**Figure 7:**
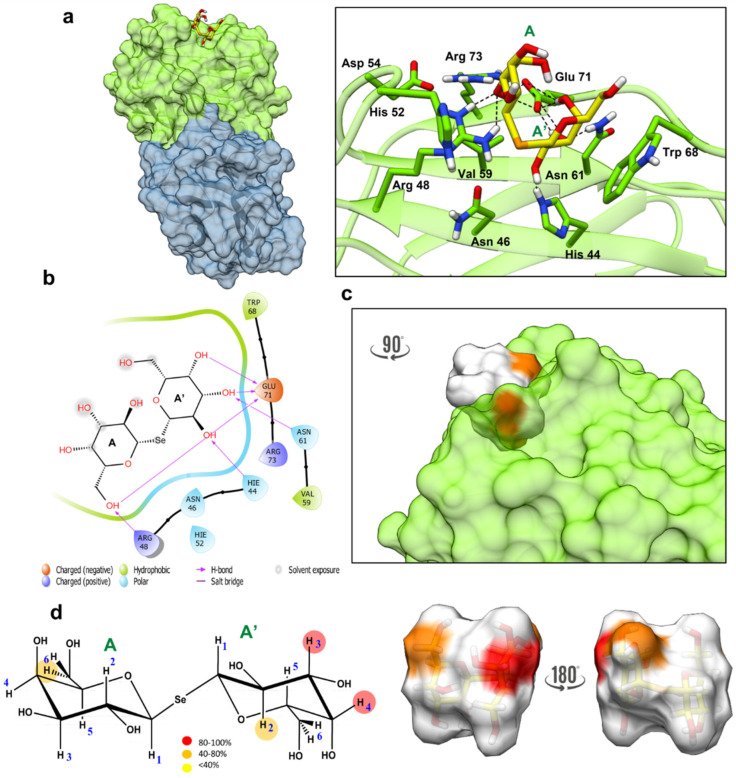
Interaction model between Gal-1 and SeDG). (**a**) The 3D model derived by docking and MD calculations of the Gal-1—SeDG system. A representative pose of the most populated MD cluster was considered to depict the interaction highlighting the main residues involved in the binding. (**b**) The 2D plot of the interactions between the SeDG and Gal-1 binding pocket residues. (**c**) Gal-1/SeDG 3D complex as derived by the combination of the MD and STD-NMR results. The ligand surface is colored according to the STD enhancements. (**d**) Interaction epitope mapping of SeDG derived from STD-NMR data on the left and a 3D representation of SeDG glycoside in the bioactive conformation, with the molecular surface colored according to the STD enhancements.

The obtained results were then used with the selenoglycosides as follows. The SeDG ligands were indeed parametrized using Gaussian 09 [19] and VFFDT [20,21] and an MD simulation of the ligand alone (free state) was performed to evaluate the stability of the ligand and possible parametrization errors. The results highlighted that in the free state, two different families for the ligand were observed, which differed in their adoption of an extended and V-shaped conformation. Furthermore, MD simulation showed that the two conformational families were similarly populated (Supplementary Materials Figure S6). The above satisfactory results permitted an MD simulation of the complexes formed by Gal-1 and Gal-3^CRD^ and the parametrized SeDG to be run, modeled in the position Sym(O) assumed in the binding pocket with the extended shape as the starting conformation (Supplementary Materials Figures S4–S6). The MD analysis of SeDG bound to Gal-1 and Gal-3^CRD^ (Figure 6 and Figure 7) provided the 3D binding profiles of the complexes and helped to understand how the ligands were accommodated into the binding pocket of the two proteins. For both systems, the root mean square deviation (RMSD) analysis showed the stability of the complex, with the ligand being stable in the binding pocket for the entire simulation time (Supplementary Materials Figure S7). 

#### 2.4.1. Gal-3^CRD^—SeDG 3D Complex

An evaluation of the most representative poses derived from the MD simulation allowed observation of how the SeDG ligand was accommodated in the Gal-3^CRD^ binding site and partially solvent exposed. The most stable interactions were established between Arg162 and the hydroxyl moiety at position 4 of the Gal-A subunit of SeDG (Figure 6) and Asn174 and 6OH of the Gal-A’ subunit. Moreover, Glu184 was an important residue as it simultaneously interacted with OH moieties at position 2 and 3 of the A subunit, and also with the OH moiety at position 6 of the A’ subunit (Figure 6a,b). His158 and Trp181 were also involved in the complex stabilization by establishing A H-bond and hydrophobic Van der Waals interaction with galactose, respectively. The water density around the binding pocket was also evaluated, as 12 water molecules have previously been reported to play key roles in mediating binding and ligand stabilization in the binding pocket [22,23]. Here, and in accordance with the literature, a stable water density was located around the ligand, and some water molecules acted as a bridge between some protein residues and the ligand, as occurred with the Arg144 and Gal-A subunit (Supplementary Materials Figure S8). 

The obtained results were further compared with the Gal-3:TDG complex (PDB ID: 4JC1). This complex proposes an internal Gal residue interacting with His158 and Arg162 through its OH moiety at position 4 and with Glu184 and Asn174 through 6OH. Moreover, the inner ring establishes a π stacking interaction with Trp181. Instead, the so-called distal residue is anchored though the hydroxyl group at position 2, which interacts with Arg162 and Glu184 [24]. This is in full agreement with our results, where the A subunit corresponds to the inner sugar unit while the A’ subunit represents the distal one. Moreover, the superimposition of both complexes shows an almost identical disposition of the ligands (Figure S9).

Combining the information derived from docking, MD simulation, and STD NMR analysis, it was possible to propose a 3D complex as represented in Figure 6c, with both Gal subunits partially involved in the binding with Gal-3^CRD^. The proposed 3D model highlighted how all protons but H1 were recognized in the A subunit while the Gal subunit A’ only showed interactions through protons H2 and H3 (Figure 6d). These data were in accordance with the ITC results, as there were a huge number of protons involved in the interaction, and no big conformational rearrangement as the initial extended conformation was maintained (compared to Gal-1, see below) [25].

#### 2.4.2. Gal-1–SeDG 3D Complex

As in the case of Gal-3^CRD^, MD simulations were also used to evaluate how SeDG interacted with Gal-1. The computational analysis highlighted how SeDG was differently accommodated in Gal-1 binding and adopted a different conformation if compared with Gal-3. Indeed, the data showed how SeDG adopted the V-shaped conformation, stabilized by Van der Waals interactions between A and His52 and A’ and Trp68. An important stabilizing interaction involved 6OH of subunit A with Arg48 and Glu71, with 6OH acting as an important anchoring point through a stable polar interaction. If Gal subunit A remained quite stable in the binding site during the MD simulation, a higher flexibility would be found for Gal subunit A’ of SeDG. The most stable H-bond interaction in Gal-A’ was established between Asn61 and 3OH (Figure 7a). Indeed, Gal-A’ hydroxyl moieties at positions 2, 3, and 4 showed transient interactions with Glu71 during the MD simulations, and 2-OH Gal-A’ and His44. Therefore, the above information highlighted that for galactose subunit A, the interaction occurred only through 6OH, which acted as an anchor while, for galactose A’, the interaction took place trough hydroxyl moieties 2, 3, and 4 (Figure 7b). In addition, a deep inspection of the SeDG–Gal-1 3D complex showed how neither H1 nor H5 were involved in the interaction, with it being possible to propose the epitope mapping represented in Figure 7c,d.

The complex was again compared with the Gal-1:TDG system (PDB ID: 3OYW). In this case, the TDG system interacts with Gal-1 mainly through hydrogen bonds with 4OH and 6OH, a stacking interaction with Trp68 and a van der Waals contact between His52 and the sulphur atom. Meanwhile, the distal subunit interacts only through the hydroxyl moiety at position 2 [26]. Therefore, when comparing the Gal-1:TDG with our system, the first observable difference is the ligand conformation. For the selenoglycoside, the ligand adopts a V-shaped conformation, differently accommodating the ligand with most interactions involving residues of the S1 inner saccharide (galactose A’) while the distal Gal A is anchored through the 6OH (Supplementary Materials Figure S10).

Furthermore, the proposed 3D complex is in agreement with the ITC results as the number of protons involved in the interaction was lower than in Gal-3^CRD^.

## 3. Materials and Methods

### 3.1. Protein and Molecule Production

Human galectin-3^CRD^ (named Gal-3^CRD^) was produced in Escherichia coli according to previous studies [10]. The human gene gal1 (named Gal-1) was cloned in pETM11 plasmid and the his-tagged protein (residues 1–135) was produced in E. coli strain BL21(DE3)GOLD. Gal-1 expression was carried out in growing cells at 37 °C and the protein expression was induced by the addition of 0.5 mM isopropyl β-D-1-thiogalactopyranoside (IPTG). The induction was protracted at 22 °C for 16 h. Recombinant proteins were purified by a two-step purification procedure consisting of Ni^2+^-affinity and size-exclusion chromatography. Both proteins were homogeneous. Purified proteins were, finally, stored in gel-filtration buffer (20 mM Tris-HCl pH 7.5, 150 mM NaCl, and 1 mM 1,4-dithiothreitol (DTT)). 

SeDG and unsymSeDG synthesis were previously reported in Di Gaetano et al., 2019 and 2022 [9,27], respectively.

### 3.2. Circular Dichroism Analyses

The CD spectra were measured on a Jasco J-810 spectropolarimeter equipped with a Peltier thermostatic cell holder. The measurements were performed at 20 °C using a 0.1-cm path length cell in 10 mM sodium phosphate, 1 mM DTT, pH 7.4. Far-UV CD spectra were monitored from 195 to 260 nm using Gal-1 and Gal-3^CRD^ final concentrations of 10 μM. Thermal denaturation was performed by fixing the CD signal at 218 nm and increasing the temperature from 20 to 90 °C (slope 1 °C·min^−^^1^). CD spectra were averaged over at least three independent scans and the baselines corrected by subtracting the buffer contribution [28,29].

### 3.3. Light Scattering Analyses

Static light scattering experiments were carried out using a MiniDawn Treos spectrometer (Wyatt Technology, Santa Barbara, CA, USA) connected to an AKTA Purifier FPLC System [30]. The miniDAWN TREOS system uses a laser operating at 658 nm and 3 photodetectors, enabling simultaneous measurements at angles typically between 45 and 135°. In total, 1 mg of purified protein (Gal-1 and Gal-3^CRD^) was loaded on a Superdex 75 10/300 column, equilibrated in 20 mM Tris-HCl pH 8.0, 200 mM NaCl, and 1 mM DTT. A flow rate of 0.5 mL × min^−1^ was applied. Elution profiles were detected by a Shodex interferometric refractometer and data were analyzed using ASTRA 5.3.4.14 software (Wyatt Technology, Santa Barbara, CA, USA).

### 3.4. Isothermal Titration Calorimetry (ITC)

The interactions between the molecules SeDG and unsym(Se) with Gal-1 and Gal-3^CRD^ were performed by isothermal titration calorimetry (ITC) experiments [31]. The analyses were carried out on an ITC200 calorimeter (MicroCal/GE Healthcare, Boston, MA, USA) at 298 K in 20 mM NaP, 150 mM NaCl pH 7.5 buffer. SeDG and unsym(Se) (500 μM) were dissolved in the same protein buffer and were titrated into a solution of Gal-1 or Gal-3^CRD^ (20 μM). A solution of 39.4 μL of the ligands was titrated in aliquots of 1.5 μL into a cell containing 270 μL of protein. Injections were performed every 150 s, for a total of 27 injections (0.4 μL for the first injection), with a 1000 rpm stir speed. Control experiments were carried out by performing identical injections of SeDG and unsym(Se) into the cell containing buffer without protein. The binding stoichiometry, enthalpy, and equilibrium association constants were determined by fitting the corrected data to a one-set-of-site-binding model with MicroCal analysis software (GE Healthcare) and are summarized in Table 1.

### 3.5. NMR Analysis

The NMR experiments were recorded on a Bruker AVANCE NEO 600 MHz equipped with a cryo probe and data acquisition and processing were performed with TOPSPIN 4.1.1 software. Samples were prepared in 50 mM deuterated phosphate buffer (NaCl 140 mM, Na_2_HPO_4_ 10 mM, KCl 3 mM, pH 7.4) and [D4] (trimethylsilyl) propionic acid, and sodium salt (TPS 10 μM) was used as the internal reference for the spectra calibration. Data acquisition and processing were analyzed using TOPSPIN 3.2 software. 

STD NMR experiments were acquired with 32 k data points and zero-filled up to 64 k data points prior to processing. The protein resonances were selectively irradiated using 40 Gauss pulses with a length of 50 ms, setting the off-resonance pulse frequency at 40 ppm and the on-resonance pulse at 0 and 7.5 ppm: D1 3 s. An excitation sculpting with gradient pulses (esgp) was applied for the suppression of water signals. The % STD displayed in the ligands’ epitope maps were obtained by the ratio of the STD signals in the STD spectra (I0—Isat) and each relative peak intensity of the unsaturated reference spectrum (off-resonance, I0), at a saturation time of 2 s. The highest STD signal was set to 100% and all the other STDs were normalized to this value. STD NMR spectra were acquired with a protein:ligand ratio of 1:40 for Gal-3^CRD^ and 1:30 for Gal-1, with the on-resonance pulse at 0 ppm and the off-resonance at 40 ppm. Using these conditions, no STD signals were observed in the control STD NMR spectrum for the ligand alone. 

Homonuclear 2D NOESY and ROESY experiments were carried out on the ligands in the free and bound states using data sets of 2048 × 512 points and mixing times of 300–500 ms.

### 3.6. Docking Calculations

Docking calculations of all the systems were performed using AutoDock 4.2.2 and analyzed with AutoDock Tools [17]. The ligand was prepared for the docking calculations using AutoDockTools, La Jolla, CA, setting all rotatable bonds free to move during the calculations except for the glycosidic bonds. Analysis of the docking poses was performed with AutoDockTools. The grid point spacing was set at 0.375 Å, and a hexahedral box was built with x, y, and z dimensions of 40, 40, and 40 Å centered in the centroid position among the binding pocket of the Gal-1 and Gal-3^CRD^ residues. A total of 200 runs using the Lamarckian Genetic algorithm were performed, with a population size of 100, and 250,000 energy evaluations. After docking, the 200 poses were clustered in groups with a root mean square deviation less than 2.0 Å. The clusters were ranked according to the lowest energy representative of each cluster.

### 3.7. Se Parametrization

The β-Gal residue with the selenium atom (instead of an oxygen atom) in position 1 was built with Gaussian 09 [19], performing the Restrained ElectroStatic Potential (RESP) charges calculation with a Hartree-Fock calculation and a 6–31G* basis set. VFFDT [20,21] Antechamber, San Francisco, CA [32] and xLeap were combined to generate the .prep and .frcmod files. 

### 3.8. MD Simulations

Molecular dynamic calculations were performed with AMBER 18 software, San Francisco, CA in explicit waters using AMBER ff14SB, Glycam06j-1, and TIP3P force fields for the protein residues, the saccharide ligand, and the water solvent molecules, respectively. Moreover, a glycam adapted force field for the Se atom was prepared for the seleoglycosidic linkage. The ligands were built with the glycam website (https://www.glycam.org (accessed on 23 May 2021)) [33] builder utility and modified with Gaussian 09 to introduce the Se atom. For the protein preparation, missing hydrogen atoms were added, and the protonation state of ionisable groups and cap termini were computed using Maestro Protein Preparation Wizard [34]. Both systems were hydrated with an octahedral box containing the explicit TIP3P water molecules buffered at 10 Å, also adding counterions to neutralize the system. The input files were generated using the tleap modules of the AMBER package, the minimization steps were performed using the Sander module, and molecular dynamic calculations were performed using the PMEMD module. At this point, an energy minimization process was performed to refine the initial structure. The calculations employed SHAKE for the C-H bonds and 1 fs of the integration step. Periodic boundary conditions were applied and the smooth particle mesh Ewald method was used to represent the electrostatic interactions, with a grid space of 1 Å. The system was minimized, at first, holding the complex, while a second minimization was performed on the entire system. Furthermore, the whole system was slowly heated from 0 to 300 K, applying a weak restrain on the solute. Temperature was increased from 0 to 100 K at a constant volume. Then, temperature was increased from 100 to 300 K in an isobaric ensemble. Thereafter, temperature was kept constant at 300 K during 50 ps with progressive energy minimizations and solute restraint. Once completed, the restraints were removed, and the systems then advanced in an isothermal-isobaric ensemble along the production. The system coordinates were saved and used for the 100-ns simulations using the PMEMD module implemented in AMBER. Coordinate trajectories were recorded each 2 ps throughout the production runs, yielding an ensemble of 10,000 structures for each complex, which were finally analyzed. Trajectories were analyzed using the ptraj module within AMBER 18 and the VMD [35] program was used to visualize the MD results. Each trajectory was submitted to cluster analysis with respect to the ligand RMSD using the K-mean algorithm implemented in the ptraj module. The representative structure of the most populated cluster was considered to depict the complexes’ interactions. The determination of hydrogen bonds was calculated using the CPPTAJ module in AMBER 18 [32]. The h-bond is defined as occurring between an acceptor heavy atom A, a donor hydrogen atom H, and a donor heavy atom D. The distance cut-off was set to 3 Å and the A-H-D angle cut-off was 135°.

## 4. Conclusions

Galectins are a family of carbohydrate recognition proteins involved in several biological processes, such as in the modulation of signaling and cell–environment interactions, giving them roles in several diseases such as cancer and idiopathic pulmonary fibrosis. Thus, the development of novel galectin inhibitors with high affinity and high selectivity is important to allow the targeting of such diseases [36,37]. Most existing galectin inhibitors have a disaccharide scaffold, with the most selected being the thio-digalactoside (TDG), as evidenced by TD139, an ideal example in which the introduction of 4-fluorophenyl-triazole to the C3- and C3′ positions of TDG led to an ~1000-fold higher affinity for Gal-3, as compared to TDG (K_D_ = 0.068 and 75 µM measured by isothermal titration calorimetry (ITC) for TD139 and TDG, respectively) [1]. In addition, the TD139 analogue equipped with two coumarylmethyl substituents at both the C3 and C3′ positions [1,37] displayed an enhanced binding affinity for Gal-3 (K_D_ = 91 nM) 176-fold higher than that for Gal-1 (K_D_ = 16 µM). Moreover, there has been recent success in the development of single-galactoside inhibitors such as α-aryl thioglycosides. In particular, aminopyrimidine-derived galactosides were characterized as good Gal-3 inhibitors with affinities up to 1.7 μM and more than 300-fold selectivity over Gal-1 [36].

Here, we report on the study of the thermodynamic parameters and structure and dynamics of the interaction between a selenoglycoside, SeDG, and Gal-1 or Gal-3^CRD^ at the molecular level by biophysical, spectroscopic, and computational studies. In particular, NMR-obtained molecular binding information was integrated with biophysical methods, in particular CD and ITC, in order to obtain a complete picture of the complexes and insights into the design of selective inhibitors. This combined approach allowed us to highlight the different accommodation of the SeDG toward Gal-1 and Gal-3^CRD^. Thus, our data clearly showed how the interaction of SeDG with Gal-1 requires a V-shaped ligand, favored by Van der Waals interactions of the Gal-A residue and His52 and Gal-A’ and Trp68 (Figure 7 and Figure S5). Interestingly, during the MD simulation, the ligand anchored through Gal-A explored a first extended shape conformation before adopting the final stable V-shaped conformation in the binding site. Moreover, the presence of the axial-oriented hydroxyl moiety position 4 of Gal-A’ seems important as its interaction with Glu71 stabilized the V-shaped conformation. As for Gal-3^CRD^ and SeDG, the ligand instead adopted a more extended conformation, and the axial-oriented 4OH of Gal-A’ also played an important role, as it was spatially correctly positioned for the interaction with Arg144 through a water bridge (Figure 6, Figures S5 and S6). Recently, only the binding between Gal-3^CRD^ and two Se-containing Gal-3 inhibitors, specifically SeDG and (di-D-galactopyranosyl) diselenide (DSeDG) analogue with a diselenide bond between the two sugar units, was reported, showing that DSeDG displayed less affinity than SeDG. By means of MD simulations, the difference in the binding of DSeDG and SeDG with Gal-3 was justified by different energetic contributions to the binding enthalpies due to electrostatic interactions and polar solvation terms [12]. Such data was confirmed by our results obtained with SeDG. Another important point is given by the absence of binding of both Gal-1 and Gal-3^CRD^ with unsym(Se), providing an important clue for specific inhibitors toward galectins. In particular, the synthesis of the unsymmetrical selenide containing a galactose and *N*-acetyl glucosamine, resulting in a lack of interaction for both galectins, highlighted the importance of selenium, which is able to distort the bond and impede the complex formation despite the presence of the galactose.

In addition, altogether, our results indicate once more that selenium-containing carbohydrate inhibitors represent a realistic possibility of becoming novel hydrolytically stable scaffolds for a new class of galectin inhibitors. Comparison of the interaction and binding modes of SeDG to Gal-1 and Gal-3^CRD^ aims to identify hot spots to guide the rational design of inhibitors able to discern between the two galectins. Our detailed description of the 3D complexes of Gal-1 and Gal-3^CRD^ with SeDG ligands sparks the development of tailored synthetic inhibitors and therapeutics, exploiting the difference in the conformation, rigidity, and shape adopted by SeDG when in the binding site.

## Figures and Tables

**Table 1:** Static light scattering analysis of Gal-1 and Gal-3.

Protein	Theoretical MW ^a^	Experimental MW	Ratio Exp/Theor
Gal-1	17842 (Da)	35684 ± 105 (Da)	1.98
Gal-3^CRD^	18863 (Da)	19050 ± 171 (Da)	1.01

^a^ Calculated by the ProtParam tool (http://web.expasy.org/protparam/ (accessed on 23 January 2020)).

**Table 2:** Thermodynamic binding parameters from the isothermal titration calorimetry measurements of Gal-1 and Gal-3^CRD^ titrated with lactose (positive control) and SeDG at 298 K.

Protein	K_D_ (ΔM)	ΔG (Kcal/mol)	ΔH (Kcal/mol)	−TΔS (Kcal/mol)	n
Lactose					
Gal-1	45.2 ± 6.5	−5.9	−2.5 ± 1.0	−3.4 ± 0.4	0.95 ± 0.35
Gal-3^CRD^ [10]	40.8 ± 5.2	−6.1	−2.4 ± 0.9	−3.7 ± 0.3	1.03 ± 024
SeDG					
Gal-1	23.7 ± 4.2	−6.2	−2.1 ± 0.7	−4.1 ± 0.3	0.90 ± 0.32
Gal-3 [10]	21.1 ± 3.5	−6.4	−4.6 ± 0.8	−1.8 ± 0.2	0.93 ± 0.25

## Data Availability

The docking data presented in this study will be openly available in our public repository upon acceptance.

## Supplementary Materials

The following supporting information can be downloaded at: https://www.mdpi.com/article/10.3390/ijms23158273/s1.

## Abbreviations

Digalactosyl selenide	SeDG
unsymmetrical diglycosyl selenides	unsym(Se)
Isopropyl β-D-1-thiogalactopyranoside	IPTG
