# Supplementary material for: Exploring the Molecular Interactions of Symmetrical and Unsymmetrical Selenoglycosides with Human Galectin-1 and Galectin-3
Source: Int J Mol Sci. 2022 Jul 27;23(15):8273. doi: 10.3390/ijms23158273 (PMC9368490; doi:10.3390/ijms23158273)
Supplement: Supplementary file 1 [file ijms-23-08273-s001.zip › ijms-1803069-supplementary.pdf]

Supplementary

Exploring the molecular interactions of symmetrical and unsymmetrical Selenoglycosides with human Galectin-1 and Galectin-3

Pirone, L.[1#], Nieto- Fabregat, F.[2#], Di Gaetano, S.[1,3], Capasso, D.[3,4], Russo, R.[1], Traboni, S.[2], Molinaro, A.[2], Iadonisi, A.[2], Saviano, M.[5], Marchetti, R. [2], Silipo, A. [2*], and Pedone, E. [1,3*]

[1]Institute of Biostructures and Bioimaging, CNR, 80134 Naples, Italy
[2]Department of Chemical Sciences, University of Naples Federico II, Via Cinthia 4,80126 Naples, Italy
[3] Interuniversity Research Centre on Bioactive Peptides (CIRPEB), University of Naples "Federico II", 80134 Naples, Italy
[4]Center for Life Sciences and Technologies (CESTEV), University of Naples "Federico II", 80145 Naples, Italy
[5] Institute of Crystallography, CNR, 81100, Caserta, Italy

* Correspondence: silipo@unina.it; empedone@unina.it

[#]These authors contribute equally to this work.

Scheme S1 Chemical structures of SeDG (a), unsym(Se) (b) and lactose (c).

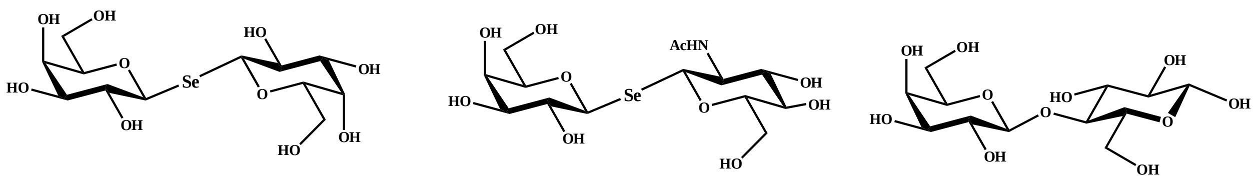

a b c

Table S1 related to Figure 6: Experimental STD epitope for SeDG bound to Gal-3^{CRD}. The T1 values of the ligand protons measured in the free state are all below 1.5 seconds. STD epitope (%): the strongest STD signal is assigned to a value of 100%, the other STD intensities were calculated accordingly. STD epitope: the absolute magnitudes of the STD signals were also reported

^{1}H	ppm	STD epitope	STD epitope (%)
**H2***	3.656	0.004623	72
**H3**	3.591	0.006442	100
**H4**	3.928	0.005248	81
**H5***	3.669	0.004623	72
**H6**	3.712	0.003160	49
**H6'***	3.651	0.004623	72

* H2, H5 and H6' signals are overlaped

Table S2 related to Figure 7: Experimental STD epitope for SeDG bound to Gal-1. The T1 values of the ligand protons measured in the free state are all below 1.5 seconds. STD epitope (%): the strongest STD signal is assigned to a value of 100%, the other STD intensities were calculated accordingly. STD epitope: the absolute magnitudes of the STD signals were also reported.

^{1}H	ppm	STD epitope	STD epitope (%)
**H2**	3.656	0.003908	63
**H3**	3.588	0.005186	83
**H4**	3.928	0.006181	100
**H6**	3.712	0.003343	54

A

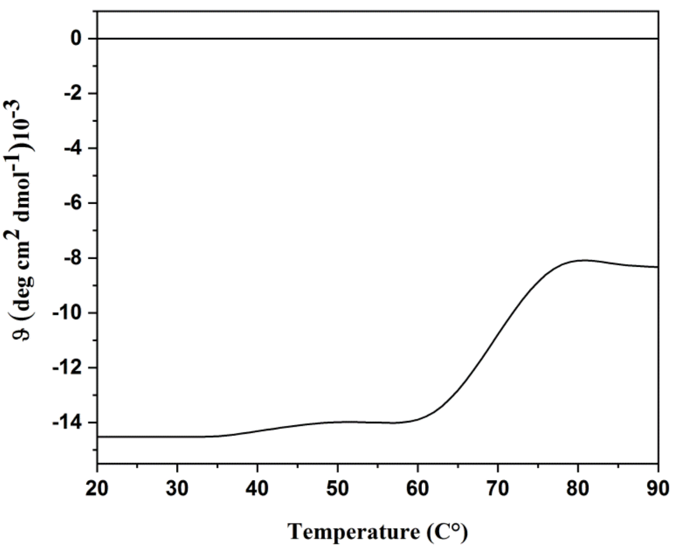

B

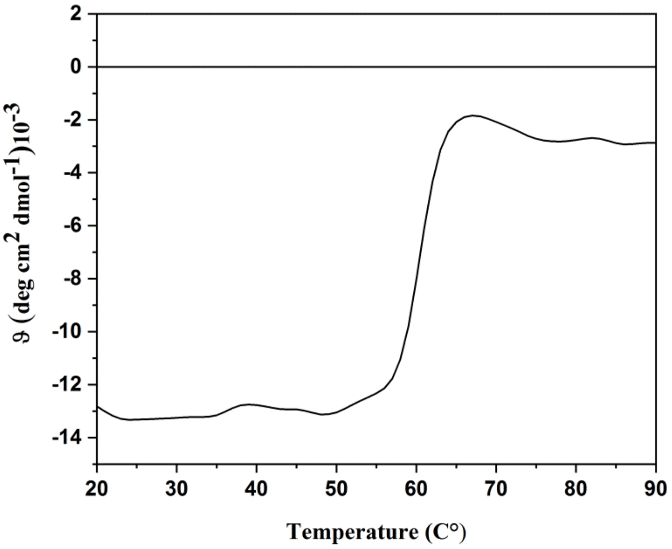

Figure S1: Thermal denaturation of A) Gal-1 and B) Gal-3 ^{CRD}: CD signal was followed at 218 nm

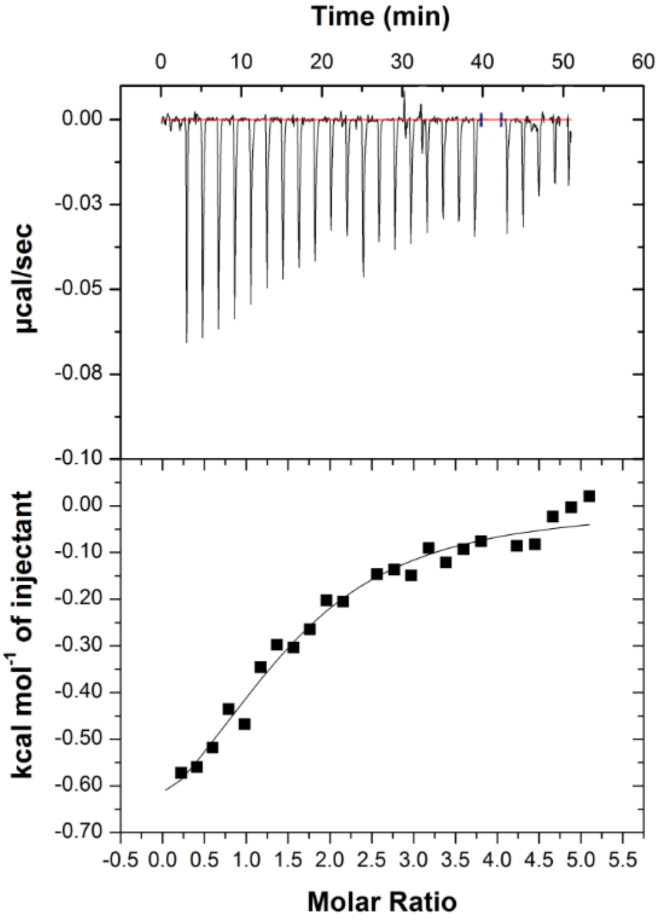

Figure S2. ITC analysis of Gal-1 binding to lactose used as control. The top and bottom panels shown raw and integrated data, respectively.

Figure S3 Curve fitting for the quantification of T1 longitudinal relaxation of SeDG protons. The data were fitted according to the following equation: f(t) = Io * [1 - a*exp (-t/T1)].

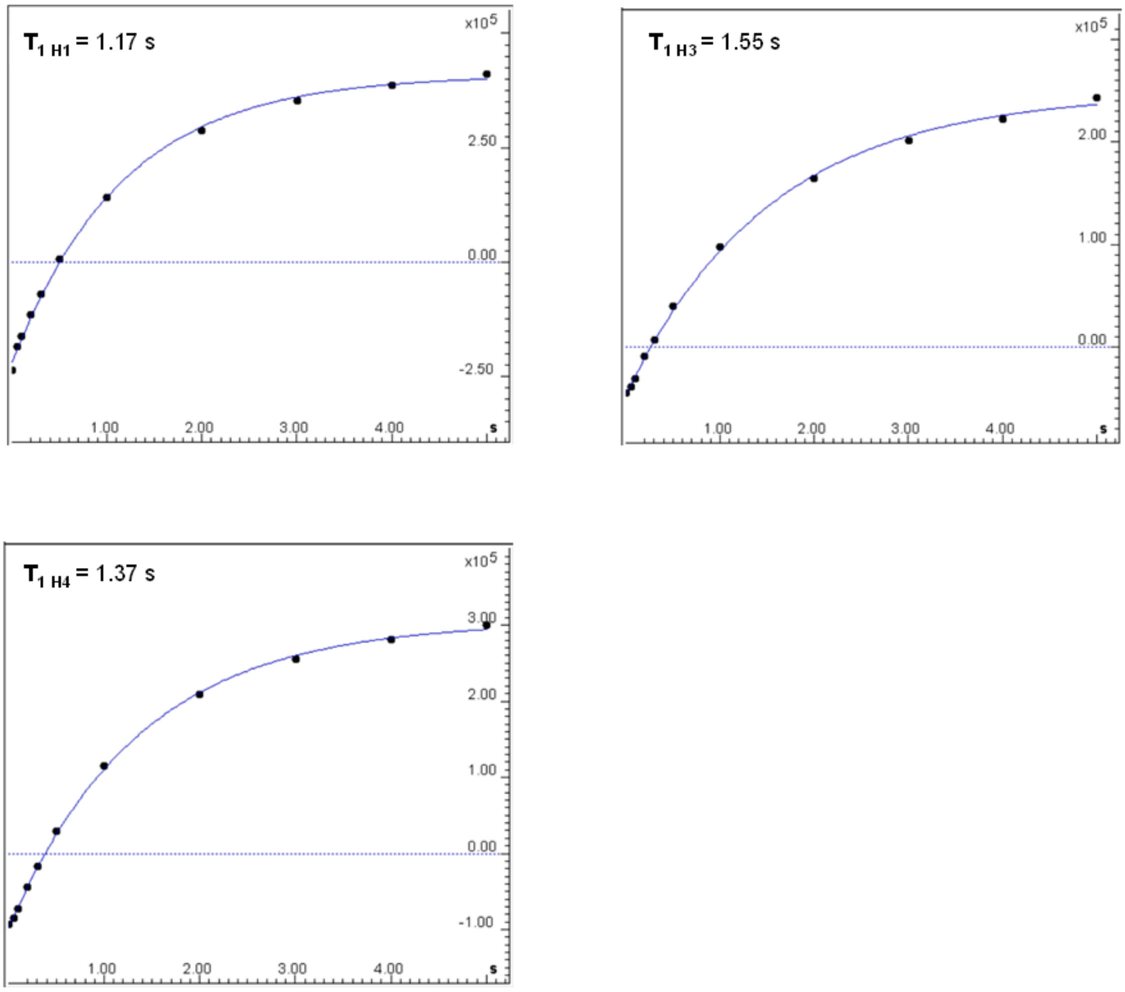

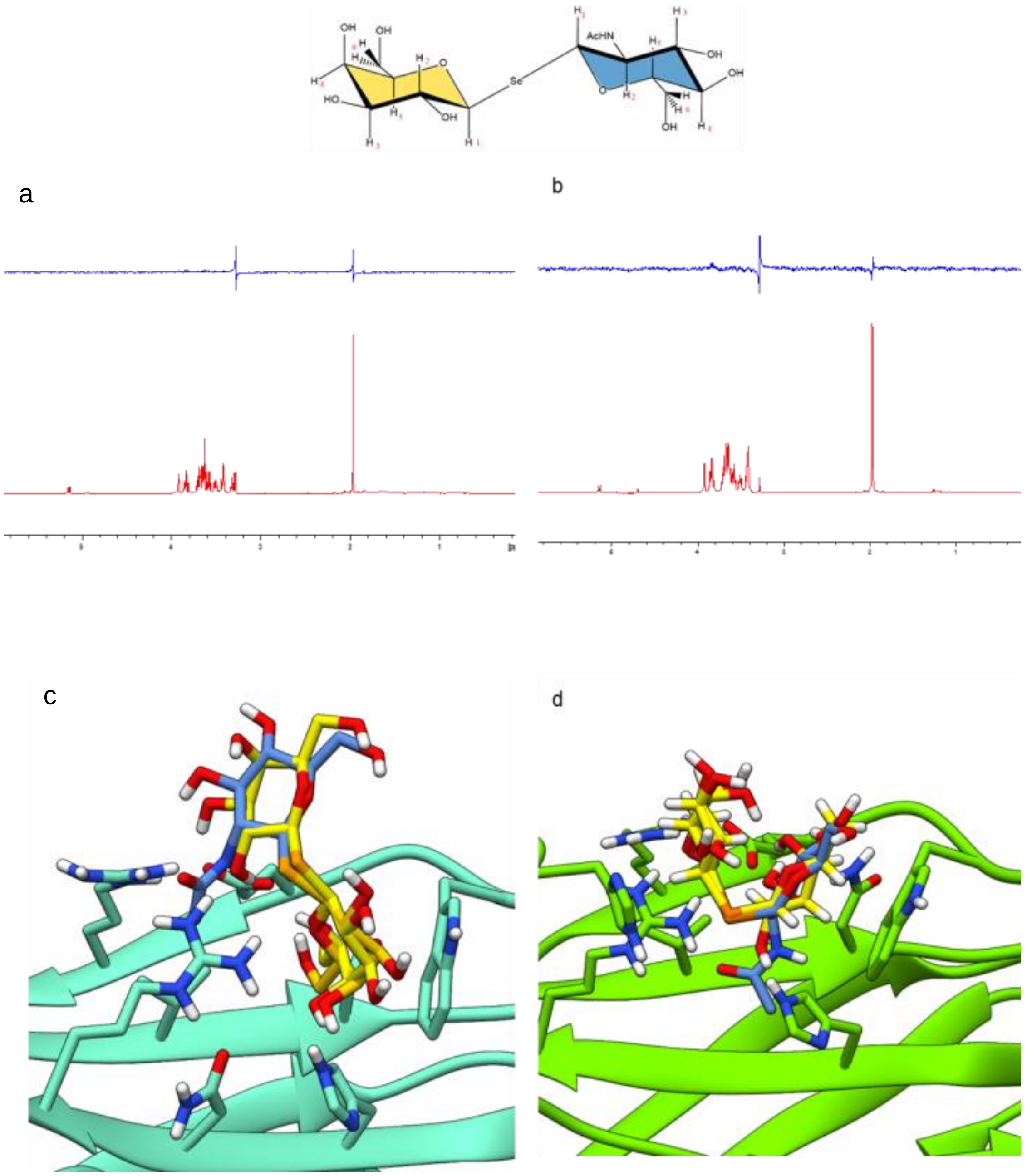

Figure S4. Unsym(Se) interaction studies. ^{1}H NMR reference spectrum (bottom) and 1D STD NMR spectrum (up) of the 1:40 mixture of Gal-3: Unsym(Se) (a) and of Gal-1: Unsym(Se) (b). Unsym(Se) ligand chemical structure was coloured according to the symbol-nomenclature for glycans (SNFG). c) 3D representativemodel of the Gal-3 – Unsym(Se) complex with the saccharidic units coloured according to SNFG nomenclature. From the superimposition with the SeDG ligand (in yellow), it is possible to see how the unsym(Se) ligand clashes with the protein because of the presence of the GlcNAc residue (in blue). d) 3D representative model of the Gal-1 – Unsym(Se) complex with the saccharidic units coloured according to SNFG nomenclature. Analogously to Gal-3 – Unsym(Se) complex, a steric clash between the protein and the N-acetyl group at position 2 of the GlcNAc moiety occurred.

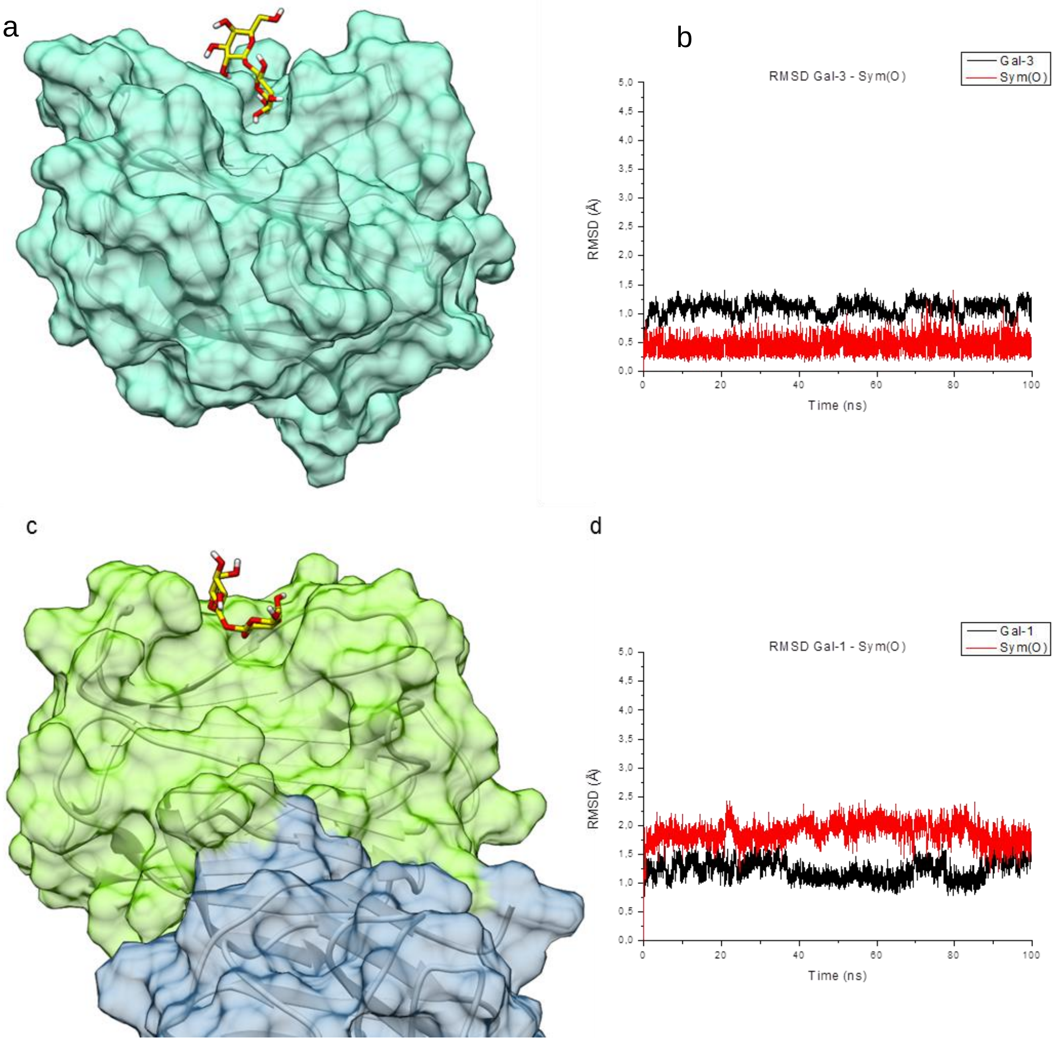

Figure S5. MD studies of Gal-1 and Gal-3 in the interaction with Sym(O) ligand. a) representative 3D model of the Gal-3 – Sym(O) complex from the most populated MD cluster. b) Root-mean square deviation (RMSD) plots of Gal-3 (black) and Sym(O) (red)calculated with respect to the protein. c) Representative 3D model of the Gal-1 – Sym(O) complex from the most populated MD cluster o. d) RMSD plots of Gal-1 (black) and Sym(O) (red)calculated with respect to the protein.

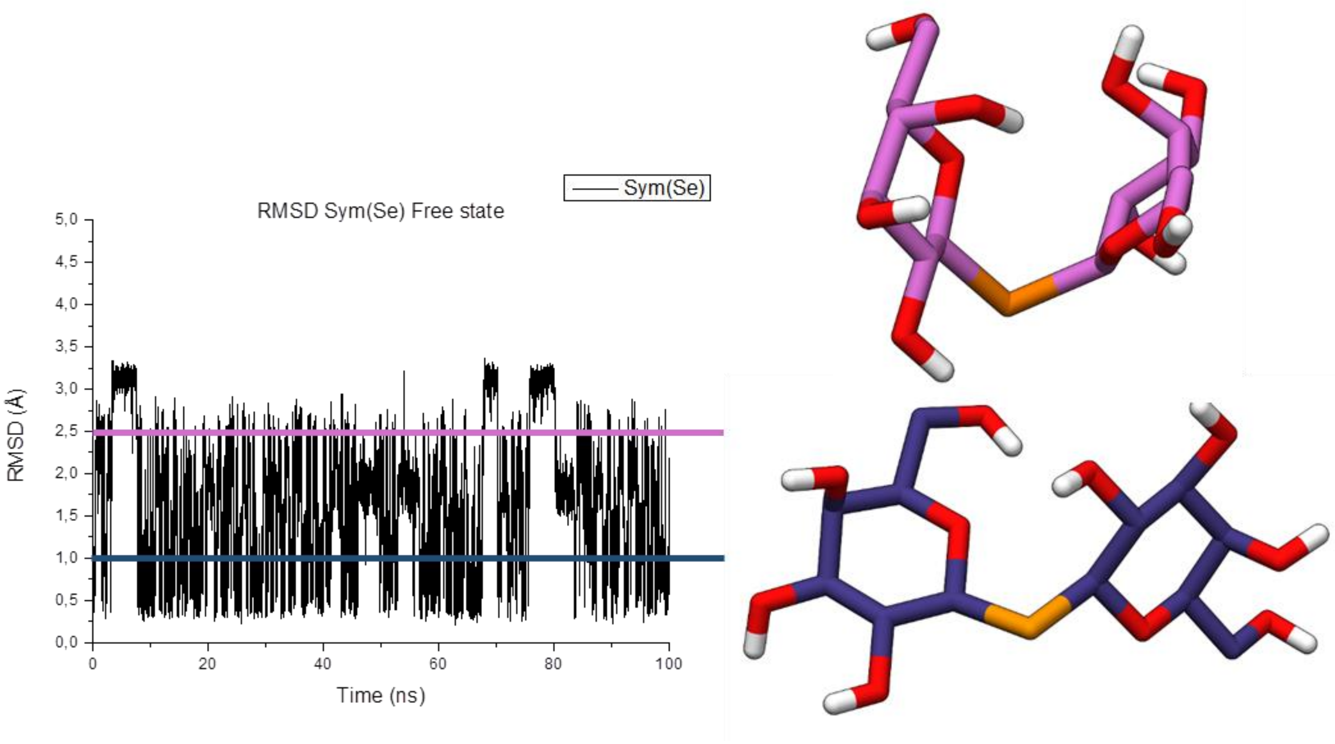

Figure S6. Analysis of SeDG in the free state. The SeDG RMSD shown two main conformational families, one with a RMSD of around 1,0 Å (dark blue, extended-shape conformation) and the second with a RMSD of2,0 Å. A third family was observed around 3,0 Å but the conformation is equivalent to that of the second family, thus, we considered a comprehensive family around 2.5 Å (purple, V-shaped conformation).

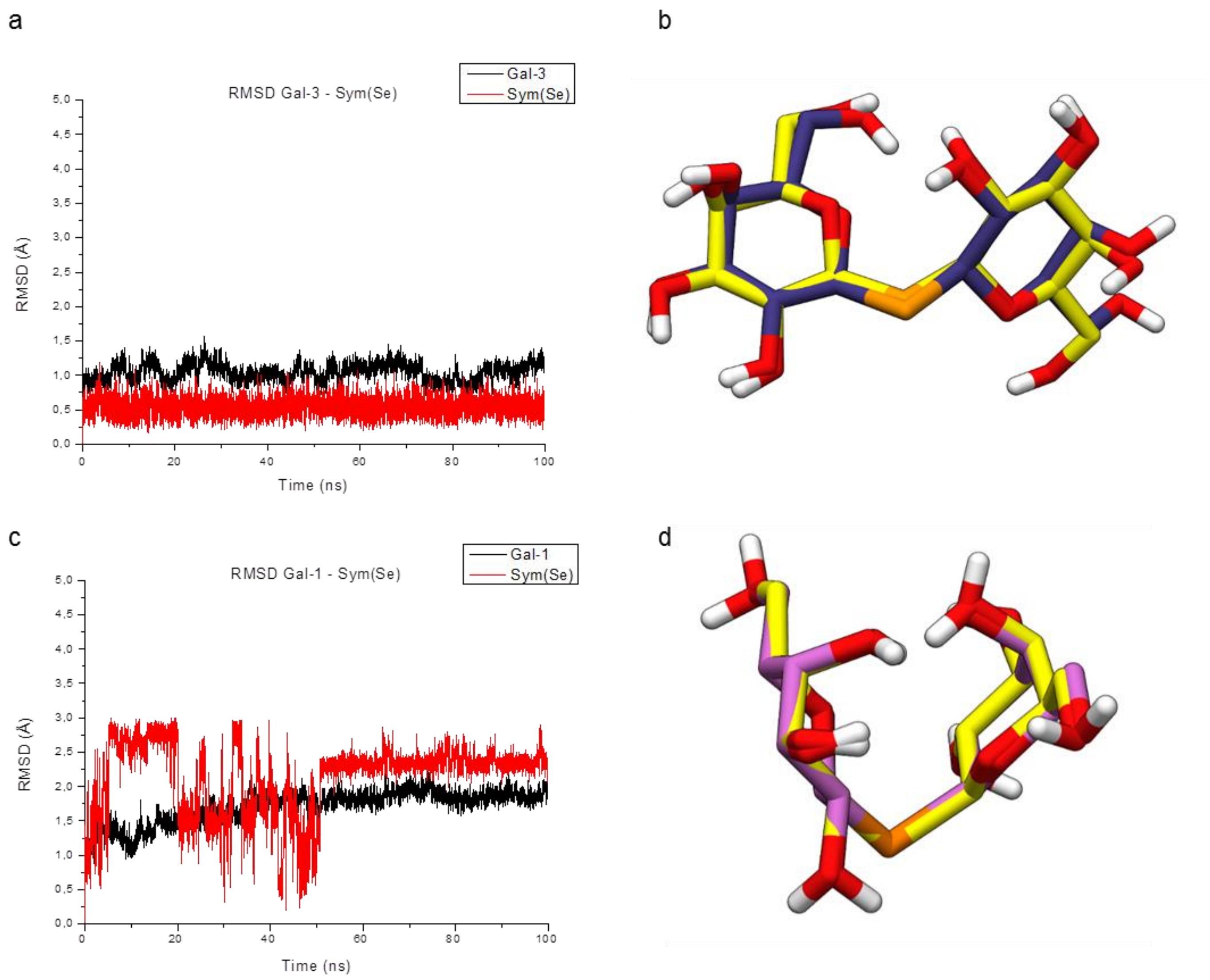

Figure S7. RMSD plots of the Gal-3/Gal-1: SeDG complexes. a) RMSD of Gal-3 (black) and SeDG (red) calculated having the protein as reference, showing the stability of both ligand and protein. The ligand exhibited only one conformational family. b) Ligand bound conformation in yellow compared with the conformation adopeted in the free state corresponding to the extended-shape in dark blue. c) RMSD of Gal-1 (black) and SeDG red calculated with respect to the protein, showing the stability of both ligand and protein. Two possible families were observed for the ligand, corresponding to the initial extended-shape and the stabilized V-shaped conformation upon the conformational change during the last 50 ns. d) Stabilized ligand bound conformationin yellow compared with the reference V-shaped conformation reference in purple**.**

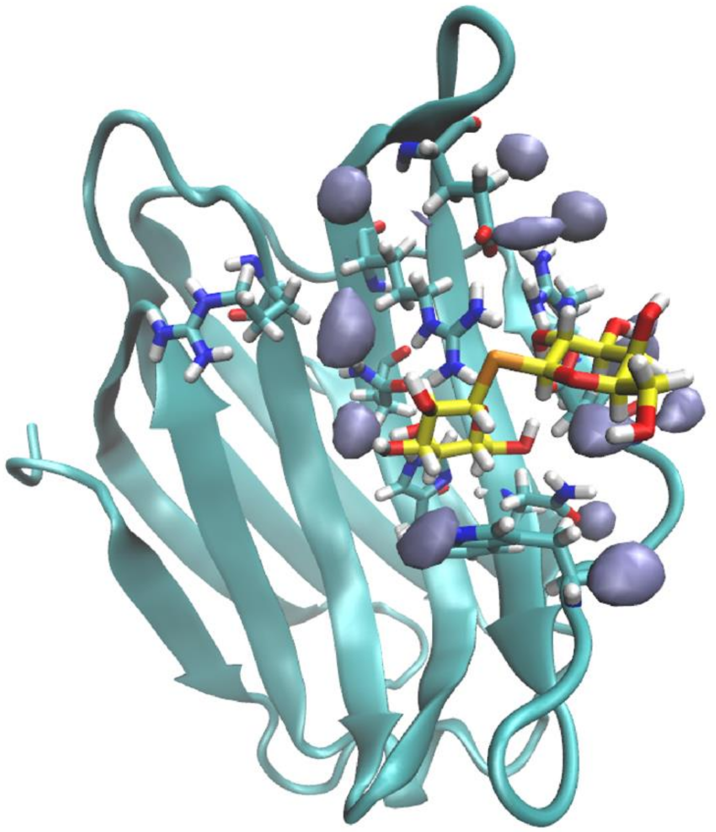

FigureS8. Water density occupancy (steel blue), calculated along the MD using VMD software, at a distance of 4 Å of around SeDG ligand and the binding pocket residues. .
